# The addition of amoxicillin improves the efficacy of the imipenem-avibactam combination against *Mycobacterium abscessus* in a mouse model of infection

**DOI:** 10.1128/aac.00534-25

**Published:** 2025-07-21

**Authors:** Vincent Le Moigne, Maria Bitar, Michel Arthur, Jean-Luc Mainardi, Jean-Louis Herrmann

**Affiliations:** 1 Université Paris-Saclay, UVSQ, INSERM, Infection et Inflammation 27048, Montigny-le-Bretonneux, France; 2 INSERM ERL 1336, UMRS 8228, Sorbonne Université-ENS-PSL-CNRS 27063, Paris, France; 3 Assistance Publique-Hôpitaux de Paris, Centre-Université de Paris, Université Paris Cité, Service de Microbiologie, Hôpital Européen Georges Pompidou 55647, Paris, France; 4 Assistance Publique-Hôpitaux de Paris, GHU Paris-Saclay, Service de Microbiologie, Hôpital Raymond Poincaré 55531, Garches, France; City St George's, University of London, London, United Kingdom

**Keywords:** cystic fibrosis, beta-lactams, DBO, *Mycobacterium abscessus*

## Abstract

*Mycobacterium abscessus* lung infections remain difficult to treat. Previous studies have shown that the combinations of a carbapenem and a second-generation β-lactamase inhibitor are effective *in vitro*, in macrophages, and in zebrafish embryos. We report here that imipenem combined with avibactam is also effective in a C3HeB/FeJ mouse model of infection in reducing bacterial burden in organs. The addition of amoxicillin improved the antibacterial activity of this combination in the lungs and kidneys.

## INTRODUCTION

*Mycobacterium abscessus* is a rapidly growing mycobacteria responsible for pulmonary diseases in patients suffering either from chronic obstructive pulmonary diseases or cystic fibrosis (1, 2). Smooth (S) variants of *M. abscessus* form biofilms in the early phases of lung colonization. The onset of acute and invasive lung infection is triggered by the S to rough (R) morphotype transition. R variants form aggregates (cords) preventing phagocytosis (3).

*M. abscessus* is resistant to a large array of antibiotics. The CF Foundation and the ECFS recommend that treatment of *M. abscessus* pulmonary infections should include an intensive phase followed by a continuation phase (4). The intensive phase should include a combination of imipenem (IPM), the currently recommended β-lactam, amikacin, azithromycin, and tigecycline during 3–12 weeks. The treatment of the intensive phase is guided but not dictated by drug susceptibility testing (4). A panel of *per os* drugs is available for the continuation phase (≥12 months) (4).

*M. abscessus* produces a broad-spectrum β-lactamase, Bla_Mab_ (5), which hydrolyzes β-lactams and is inhibited by diazabicyclooctane (DBO) β-lactamase inhibitors such as avibactam (AVI) (6). AVI should, therefore, be considered to improve the activity of β-lactam antibiotics, although it is not yet part of the recommended treatment (7, 8). DBOs restore the activity of β-lactams that are effectively inactivated by Bla_Mab_, such as amoxicillin (AMX) (6). In contrast, DBOs minimally potentiate the *in vitro* activity of carbapenems, which are ineffectively inactivated by Bla_Mab_, only leading to one- to two-fold reductions of the MICs of IPM and tebipenem against *M. abscessus* (9–17). In spite of this limited impact on MICs, DBOs were found to improve the *in vivo* activity of carbapenems in mouse and zebrafish models (9, 18, 19). Drug combinations including two β-lactams and a DBO inhibitor displayed promising activity *in vitro* (13, 20–23), but their *in vivo* evaluation remains limited (23).

Herein, we explored, in the C3HeB/FeJ mouse model of *M. abscessus* infection (24), the efficacy of IPM combined with AVI in the absence or presence of AMX. AMX was chosen because this drug, in association with AVI, is active *in vitro* against *M. abscessus* (6) and can potentially act in synergy with IPM by acting on complementary sets of targets (L,D-transpeptidases and PBPs). The ability of DBOs to protect AMX and IPM against Bla_Mab_, in combination with the potential intrinsic activities of DBOs on L,D-transpeptidase and PBP targets, supports the rationale behind the potent activity of the triple combination (25, 26). We compared the efficacy of the drug regimen against the R and S variants of *M. abscessus* strain ATCC 19977.

All animal experiments were performed according to ethical guidelines and with ethical committee (N°047 with agreement A783223) agreement APAFIS #44601-2023090514417206 v3. We first evaluated drug efficacy against the R variant of the *M. abscessus* strain ATCC 19977. Against this strain, the MICs of IPM alone, in the presence of AVI (4 µg/mL), and in the presence of both AVI (4 µg/mL) and AMX (4 µg/mL) were 4, 2, and 1 µg/mL, respectively. AVI alone did not inhibit the growth of *M. abscessus* (MICs > 256 µg/mL) (16).

**Fig 1:**
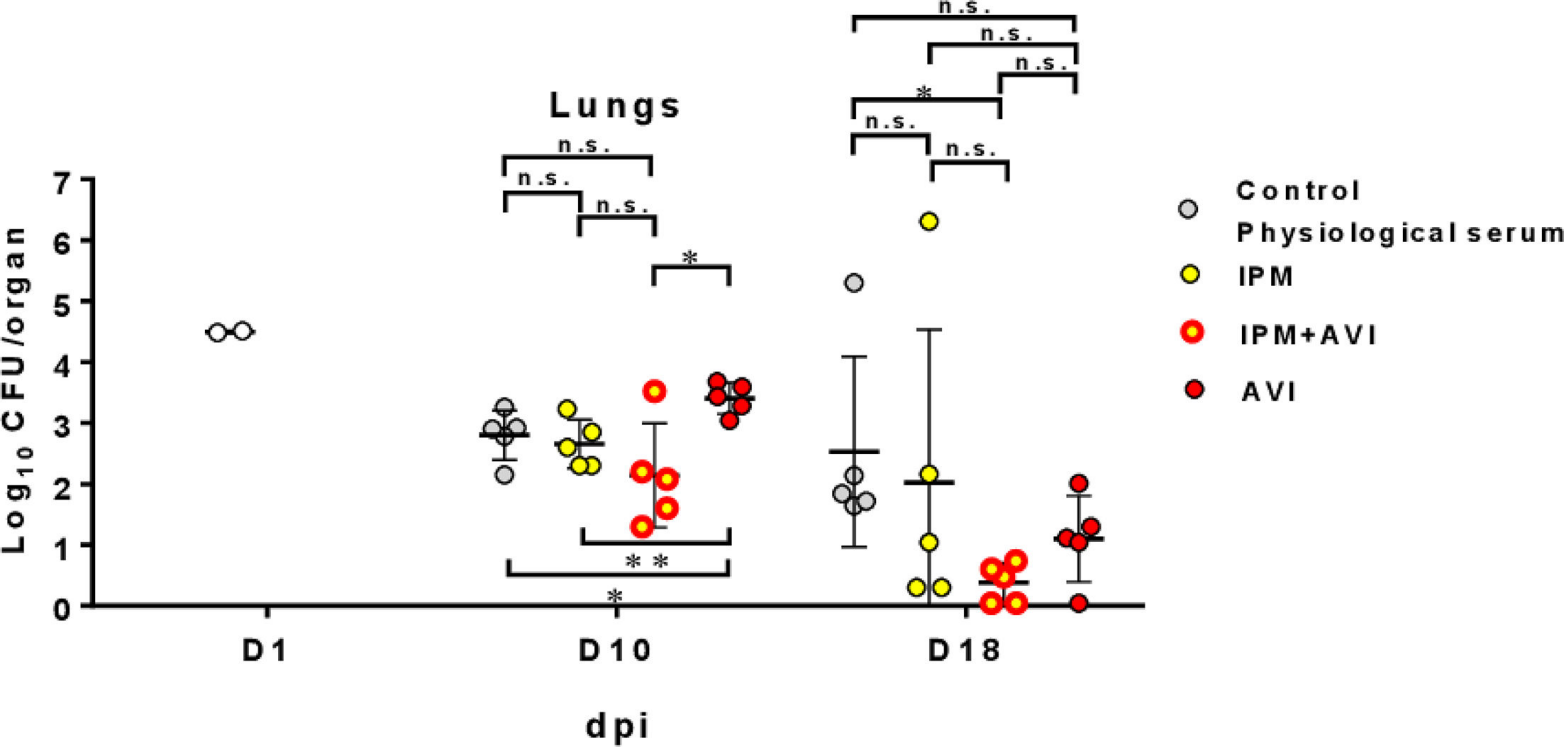
Bacterial persistence of *M. abscessus* CIP 104536^T^ rough variant in the lungs of C3HeB/FeJ mice after i.v. infection (0.6 × 10^6^ CFU/mouse) and treated with imipenem (IPM), avibactam (AVI), or the imipenem-avibactam combination (IPM-AVI). Mice were infected in the tail vein with 0.6 × 10^6^ CFU/mouse in a total volume of 200 µL of water containing 0.9% sodium chloride. The following day (before treatment started), two mice were euthanized, and lungs were harvested to determine baseline bacterial burden. Lungs were homogenized, serially diluted, and plated onto vancomycin, colistin sulfate, amphotericin B, and trimethoprim (VCAT) chocolate agar plates (BioMérieux, France) and incubated for 5–6 days at 37°C prior to CFU count. Antibiotic treatment began at 3 days post-infection (dpi). Mice were treated starting on day 3 for 5 days (D10) or 12 days (D18) by twice daily (i.e., every 12 hours) subcutaneous injections of 100 mg/kg IPM (imipenem-cilastatin; Laboratoire Arrow, France) in saline solution, of 100 mg/kg AVI (Advanced ChemBlocks, CA, USA) in saline solution, or of 100 mg/kg IPM plus 100 mg/kg AVI. All antibiotics were resuspended in sterile water, aliquoted, and frozen at −20°C until single use after defrosting. A control group received twice daily subcutaneous injections of saline. Mice were euthanized 3 days after antibiotic cessation to allow antibiotic clearance. Experimental groups of mice were evaluated for bacterial burden on days 10 and 18 as described above for day 1. *n* = 5 mice were used per experiment, and bacterial load in each group is expressed as log_10_ units of CFU (±SD) cells at 1, 10, and 18 dpi. Differences between means were analyzed by two-way ANOVA and the Tukey post-test, allowing multiple comparisons. n.s. = non-significant, *P < 0.05, **P < 0.01 . Experiment was realized once.

In lungs and compared to untreated animals, AVI alone had no activity against the R variant of *M. abscessus* both at 10 and 18 days post-infection (dpi) (Fig. 1; Table S1 for statistical evaluation). Compared to untreated animals, IPM was not effective both at 10 and 18 dpi. The combination IPM-AVI was effective in lungs at 18 dpi compared to untreated animals, but the addition of AVI to IPM did not significantly improve the activity of IPM. For other organs (Fig. S1A to C; Table S1 for statistical evaluation), significant results showed that IPM was effective in the liver at 10 dpi and in the kidneys both at 10 and 18 dpi. Addition of AVI to IPM did not significantly improve the activity of IPM in organs, except in the liver at 10 dpi. In the kidneys, the clearance of bacteria was effective with IPM and IPM-AVI, with sterilization at 18 dpi in the majority of the animals (Fig. S1C). Macroscopic examination of kidneys at 18 dpi revealed abscesses in animals treated with AVI alone as well as in untreated animals, while IPM and IPM-AVI prevented the formation of abscesses (Fig. S1D, lower panel). Treatments with IPM and IPM-AVI were associated with lower spleen weight in comparison to untreated animals at 18 dpi (Fig. S1D, upper panel).

IPM and IPM-AVI treatments resulted in very low CFU counts or sterilization in organs in mice infected with the R variant. Increasing the infectious dose with the R morphotype results in an acute infection with a massive inflammatory response and death of mice in 3–5 days (27). In contrast, increasing the infection dose is feasible for the S variant, providing additional opportunities to compare the efficacy of drug regimens. Thus, the efficacy of β-lactam combinations was next evaluated against the S variant of *M. abscessus* strain ATCC 19977 at a higher inoculum.

**Fig 2:**
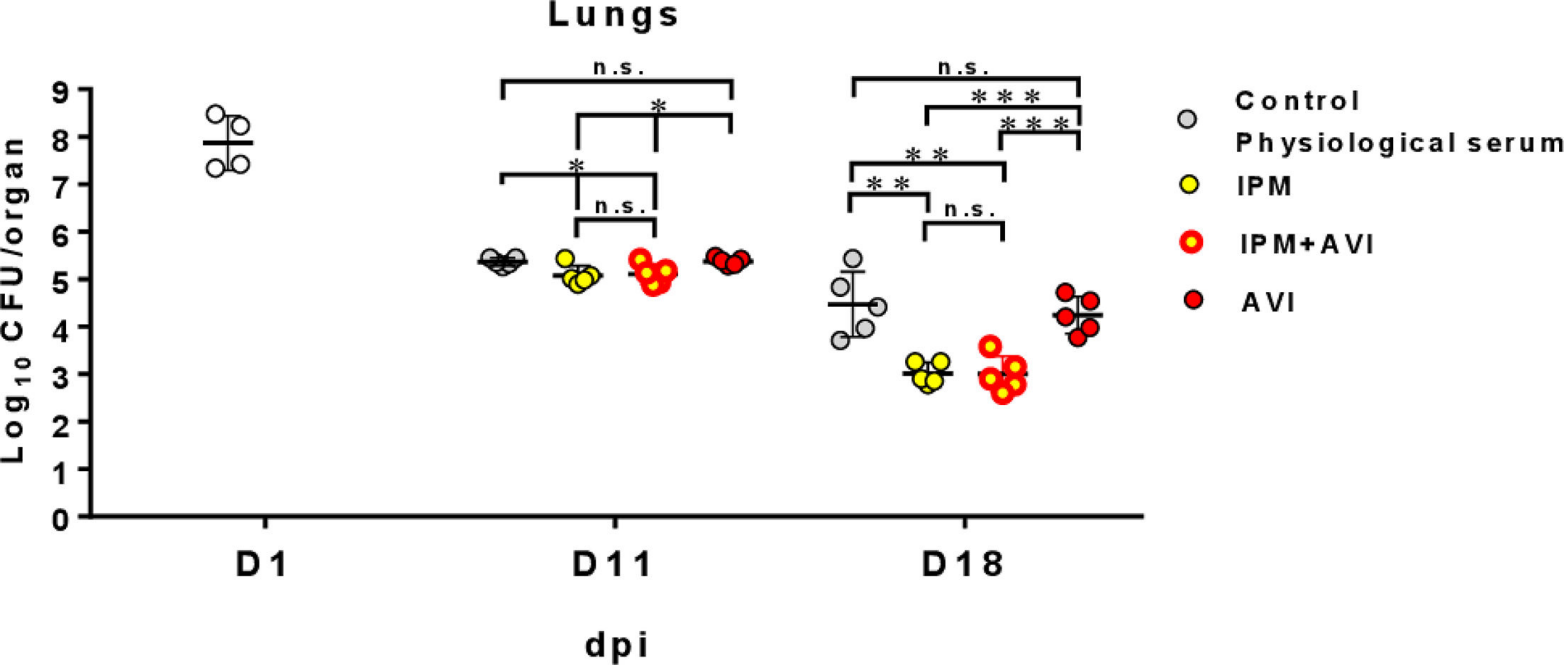
Bacterial persistence of *M. abscessus* CIP 104536^T^ smooth variant in the lungs of C3HeB/FeJ mice after i.v. infection ((9.4 × 10^7^ CFU/mouse) and treated with imipenem (IPM), avibactam (AVI), or the imipenem-avibactam combination (IPM-AVI). Bacterial persistence of *M. abscessus* CIP 104536^T^ (smooth variant) in the lungs of C3HeB/FeJ mice after infection in the tail vein with 9.4 × 10^7^ CFU/mouse in a total volume of 200 µL of water containing 0.9% sodium chloride. The following day (before treatment started), four mice were euthanized, and whole organs were harvested to determine baseline bacterial burden. CFUs in mice lungs were determined as described in previous Fig. 1. Antibiotic treatment began at 3 days post-infection (dpi). Mice were treated starting on day 3 for 6 days (D11) or 13 days (D18) by twice daily subcutaneous injections of IPM, AVI, or IPM-AVI with the same dosage (100 mg/kg) as in Fig. 1. A control group received twice daily subcutaneous injections of saline. Mice were euthanized 3 days after antibiotic cessation to allow antibiotic clearance. Experimental groups of mice were evaluated for bacterial burden on days 11 and 18 as described above for day 1. Mice (*n* = 5) were used per experiment, and bacterial load in each group is expressed as log_10_ units of CFU (±SD) cells at 1, 11, and 18 dpi. Differences between means were analyzed by two-way ANOVA and the Tukey post-test, allowing multiple comparisons. n.s. = non-significant, *P < 0.05, **P < 0.01, ***P < 0.001. Experiment was realized twice.

Against the S variant, the MICs of IPM alone, in the presence of AVI, and in the presence of AVI and AMX were 4, 2, and 0.5 µg/mL, respectively. These values were similar to those obtained for the R variant (4, 2, and 1 µg/mL). In lungs and compared to untreated animals, AVI alone had no activity (Fig. 2; Table S2 for statistical evaluation). In contrast, IPM and IPM-AVI led to significant reductions in the CFU counts both at 11 and 18 dpi. The addition of AVI to IPM did not result in any significant reduction of CFU counts. The same conclusions can be drawn from CFU counts in liver and spleen (Fig. S2A and B). In the kidneys, IPM-AVI afforded a greater reduction in CFU counts than IPM both at 11 and 18 dpi (Fig. S2C). As found for the R variant, IPM and IPM-AVI prevented the formation of abscesses at 18 dpi (Fig. S2D, lower panel). In mice infected with the S variant, the IPM and IPM-AVI regimens did not significantly reduce spleen weight in comparison to untreated animals at 18 dpi (Fig. S2D, upper panel), in contrast to what was observed for the R variant (Fig. S1D).

**Fig 3:**
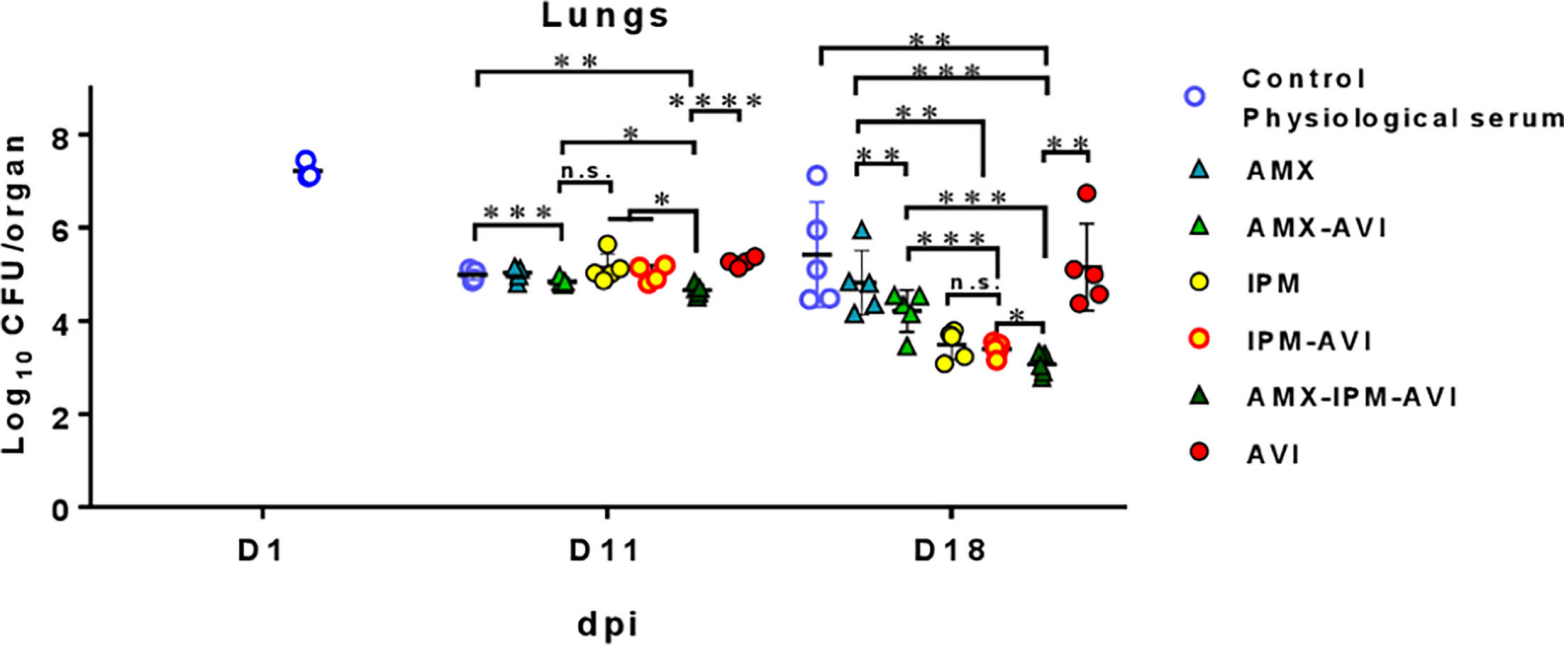
Bacterial persistence of *M. abscessus* CIP 104536^T^ smooth variant in the lungs of C3HeB/FeJ mice after i.v. infection (9.4 × 10^7^ CFU/mouse) and treated with amoxicillin (AMX), the amoxicillin-avibactam combination (AMX-AVI), imipenem (IPM), avibactam (AVI), the combination imipenem-avibactam (IPM-AVI), or the triple amoxicillin-imipenem-avibactam combination (AMX-IPM-AVI). Bacterial persistence of *M. abscessus* CIP 104536^T^ (smooth variant) in the lungs of C3HeB/FeJ mice after infection in the tail vein with 9.7 × 10^7^ CFU/mouse in a total volume of 200 µL of water containing 0.9% sodium chloride. The following day (before treatment started), three mice were euthanized, and lungs were harvested to determine baseline bacterial burden. CFUs in the lungs were determined as described in previous figures. Antibiotic treatment began at 3 days post-infection (dpi). Mice were treated starting on day 3 for 6 days (D11) or 13 days (D18) by twice daily subcutaneous injections of 100 mg/kg of AMX, IPM, or AVI in saline solution as described in previous Fig. 1 and 2 or of 100 mg/kg of each compound when double or triple combination treatments were performed, also twice daily. All antibiotics were delivered by subcutaneous administration. A control group received twice daily subcutaneous injections of saline. Mice were euthanized 3 days after antibiotic cessation to allow antibiotic clearance. Experimental groups of mice were evaluated for bacterial burden on days 11 and 18 as described above for day 1. *n* = 5 mice were used per experiment, and bacterial load in each group is expressed as log_10_ units of CFU (±SD) cells at 1, 11, and 18 dpi. Differences between means were analyzed by two-way ANOVA and the Tukey post-test, allowing multiple comparisons. n.s. = non-significant, *P < 0.05, **P < 0.01, ***P < 0.001, ****P < 0.0001. Experiment was realized once.

We next investigated whether the addition of AMX improved the efficacy of IPM and IPM-AVI against the *M. abscessus* S variant (Fig. 3; Fig. S3; Table S3 for statistical evaluation). In lungs (Fig. 3), treatment with AMX alone did not reduce the bacterial load compared to untreated mice. AMX-AVI led to significant reductions in the CFU counts only at 11 dpi (Fig. 3). IPM-AVI was more effective than AMX-AVI only at 18 dpi (Fig. 3). The AMX-IPM-AVI triple combination was more effective than IPM-AVI in reducing CFU counts both at 11 and 18 dpi.

In the kidneys, AMX-AVI led to significant reductions in the CFU counts (Fig. S3C) at both at 11 and 18 dpi. IPM was more effective than AMX-AVI (Fig. S3C) both at 11 and 18 dpi. IPM-AVI was more effective than AMX-AVI at 11 and 18 dpi (Fig. S3C). The AMX-IPM-AVI triple combination was more effective than IPM-AVI in reducing CFU both at 11 and 18 dpi. AMX-IPM-AVI and IPM-AVI were similarly effective in the liver (Fig. S3A) and spleen (Fig. S3B).

In conclusion, the IPM-AVI combination was effective in reducing bacterial load in lungs, livers, spleens, and kidneys of mice infected with the R (Fig. 1; Fig. S1) and S (Fig. 2; Fig. S2) variants of *M. abscessus*. This combination was also effective in preventing the formation of abscesses in the kidneys (Fig. S1D and S2D). Since the IPM-AVI effect seems to increase at later dpi, this reinforces the importance of timely AVI addition to protect IPM against hydrolysis (17). Addition of AMX improved the efficacy of IPM-AVI against the S variant in lungs, which is the target organ for the treatment of infections due to *M. abscessus* (Fig. 3). At 18 dpi, IPM and IPM-AVI successfully sterilized lungs and kidneys of most animals infected with the R variant. Accordingly, the benefit of the addition of AMX to the IPM-AVI combination against the R variant could not be tested in the mouse model. Another limitation concerns the dosing of the drug regimen and the difference in the pharmacokinetic parameters of the drugs in humans and mice. Indeed, if IPM serum levels achieved in mice at the dose of 100 mg/kg were similar to those obtained in humans (28), the half-life of IPM is shorter in mice than in humans. Since the antibacterial activity of IPM depends mainly on time over the MIC, the activity observed in this model is probably less than what can be achieved in humans at the recommended dose. Moreover, in humans, IPM is administered three times a day, whereas in this model, they were administered twice daily for practical reasons.

Our data indicate that triple combinations comprising IPM, AMX, and AVI are more effective than IPM combined with AVI in reducing the number of CFUs in the lungs of mice infected by *M. abscessus* (Fig. 3). Thus, the combination of available i.v. imipenem-DBO formulations with i.v. or *per os* amoxicillin should be considered by clinicians. The efficacy of this treatment should be evaluated based on the reporting of patient outcomes.

## SUPPLEMENTAL MATERIAL

Supplemental material aac.00534-25-s0001.pdf Fig. S1 to S3; Tables S1 to S3.

ASM does not own the copyrights to Supplemental Material that may be linked to, or accessed through, an article. The authors have granted ASM a non-exclusive, world-wide license to publish the Supplemental Material files. Please contact the corresponding author directly for reuse.
