# Supplementary material for: The addition of amoxicillin improves the efficacy of the imipenem-avibactam combination against Mycobacterium abscessus in a mouse model of infection
Source: Antimicrob Agents Chemother. 2025 Jul 21;69(8):e00534-25. doi: 10.1128/aac.00534-25 (PMC12327000; doi:10.1128/aac.00534-25)
Supplement: Supplemental material — Fig. S1 to S3; Tables S1 to S3. [file aac.00534-25-s0001.pdf]

Supplementary Figure 1

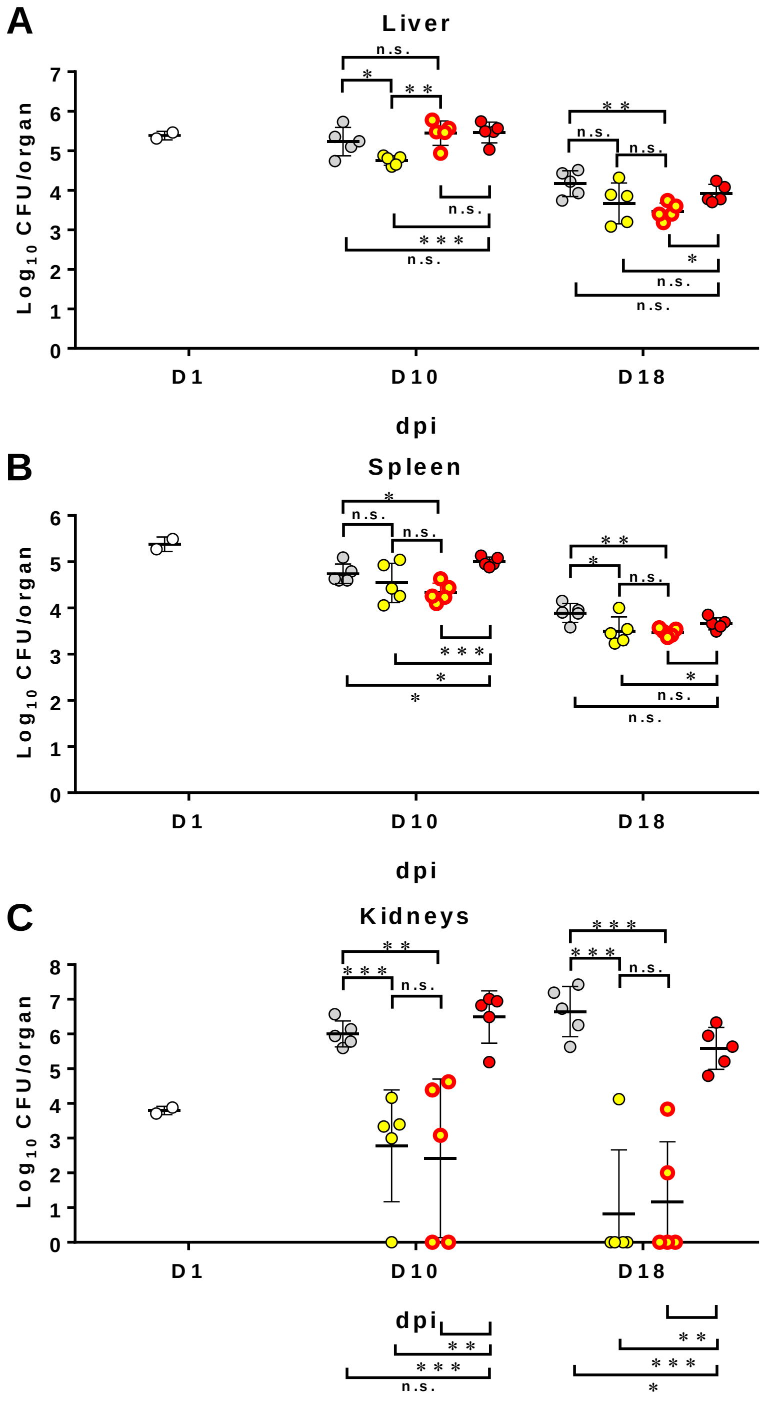

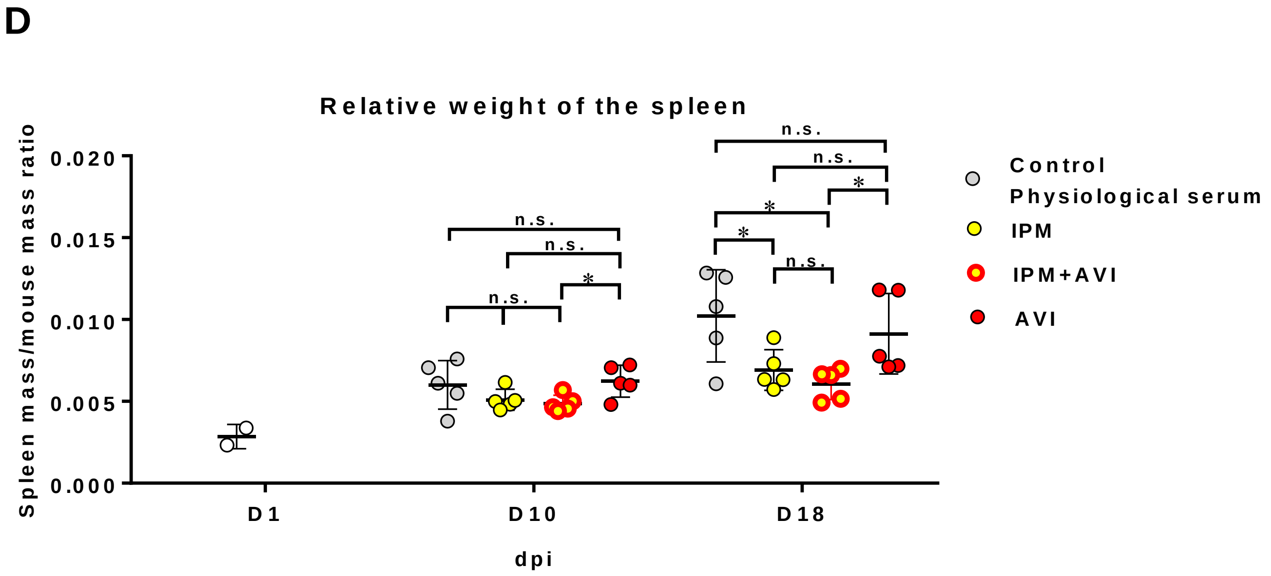

Kidneys aspect at D17

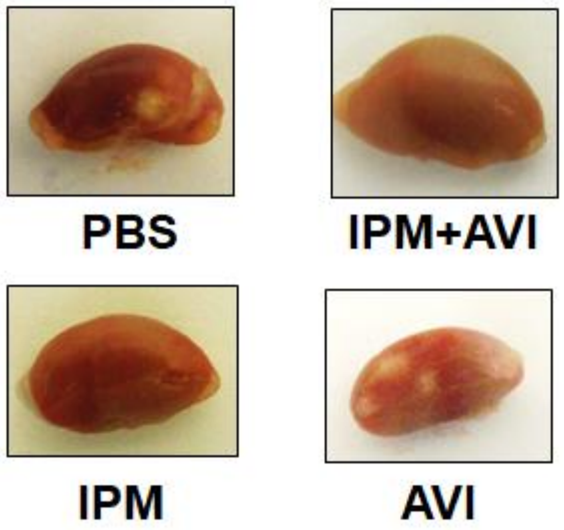

Supplementary Figure 1. Bacterial persistence in organs of *M. abscessus* rough variant in C3HeB/FeJ infected mice, relative spleen weight and kidneys aspect at 18 dpi with treaments as in Fig. 1. *M. abscessus* R-infected C3HeB/FeJ mice untreated (control PBS) or treated with imipenem (IPM), avibactam (AVI), or the IPM-AVI combination. Bacterial persistence of *M. abscessus* CIP 104536^T (rough variant) were measured in the liver (**A**), spleen (**B**) and kidneys (**C**) of C3HeB/FeJ mice after infection and treatment of mice from **Fig. 1**. Spleen masses of mice from **Fig. 1** were weighed at each time points and relative weight of spleen to each mouse weight was calculated (**D**, upper panel). Kidneys aspect at 18 dpi are presented (**D**, lower panel). Differences between means were analyzed by two-way analysis of variance (ANOVA) and the Tukey's post-test, allowing for multiple comparisons. n.s., non-significant; * $P<0.05$, ** $P<0.01$, *** $P<0.001$, **** $P<0.0001$. Experiment was realized once.

Supplementary Figure 2

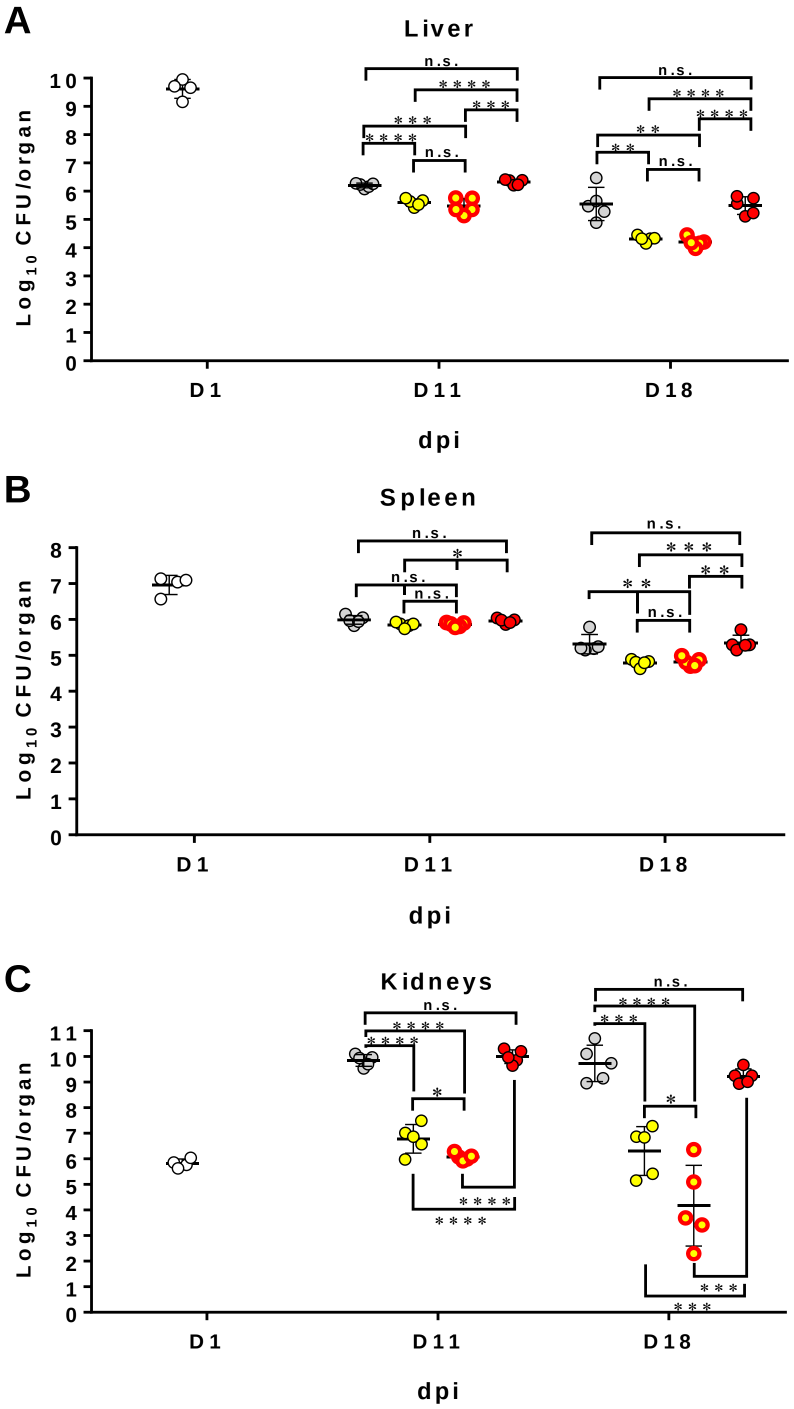

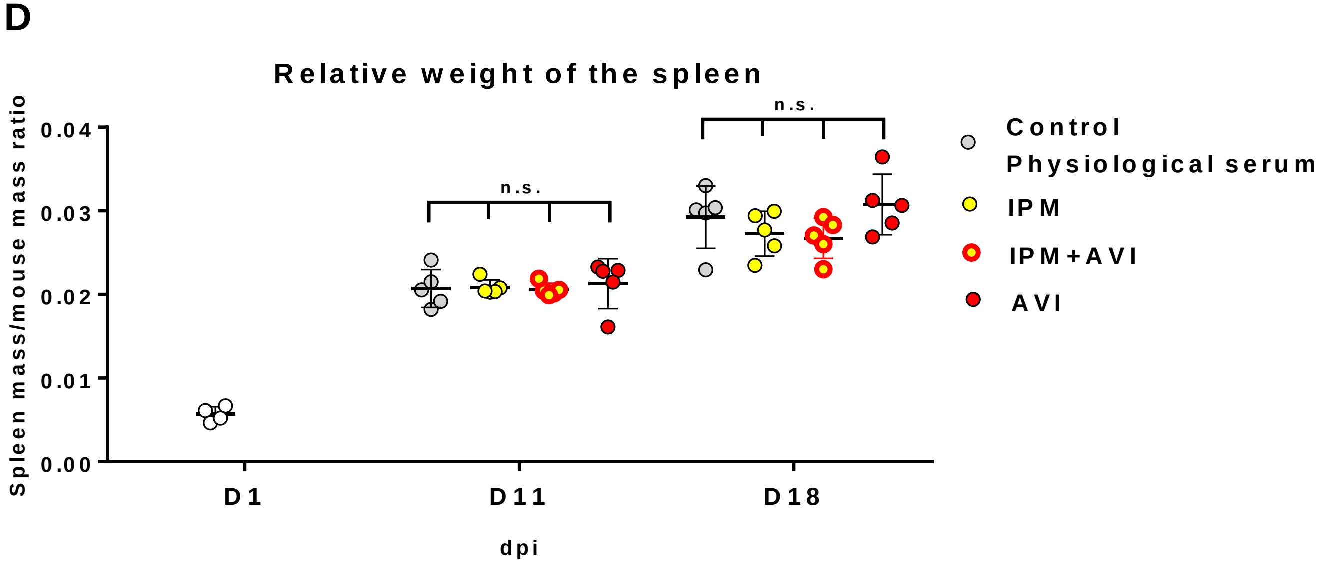

Kidneys aspect at D18

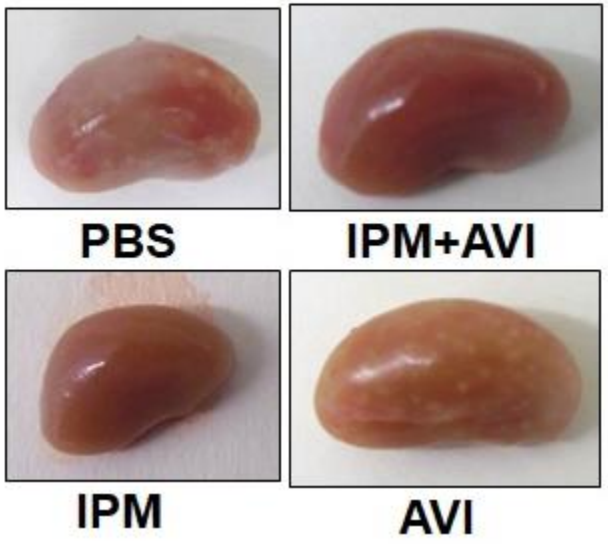

Supplementary Figure 2. Bacterial persistence in organs of *M. abscessus* smooth variant in C3HeB/FeJ infected mice, relative spleen weight and kidneys aspect at 18 dpi with treaments as in Fig. 2. *M. abscessus* S-infected C3HeB/FeJ mice untreated (control PBS) or treated with imipenem (IPM), avibactam (AVI) or the IPM-AVI combination. Bacterial persistence of *M. abscessus* CIP 104536^T (smooth variant) were measured in the liver (**A**), spleen (**B**) and kidneys (**C**) of C3HeB/FeJ mice after infection and treatment of mice from **Fig. 2**. Spleen masses of mice from **Fig. 2** were weighed at each time points and relative weight of spleen to each mouse weight was calculated (**D**, upper panel). Kidneys aspect at 18 dpi are presented (**D**, lower panel). Differences between means were analyzed by two-way analysis of variance (ANOVA) and the Tukey's post-test, allowing for multiple comparisons. n.s., non-significant; * $P<0.05$, ** $P<0.01$, *** $P<0.001$, **** $P<0.0001$. Experiment was realized once.

Supplementary Figure 3

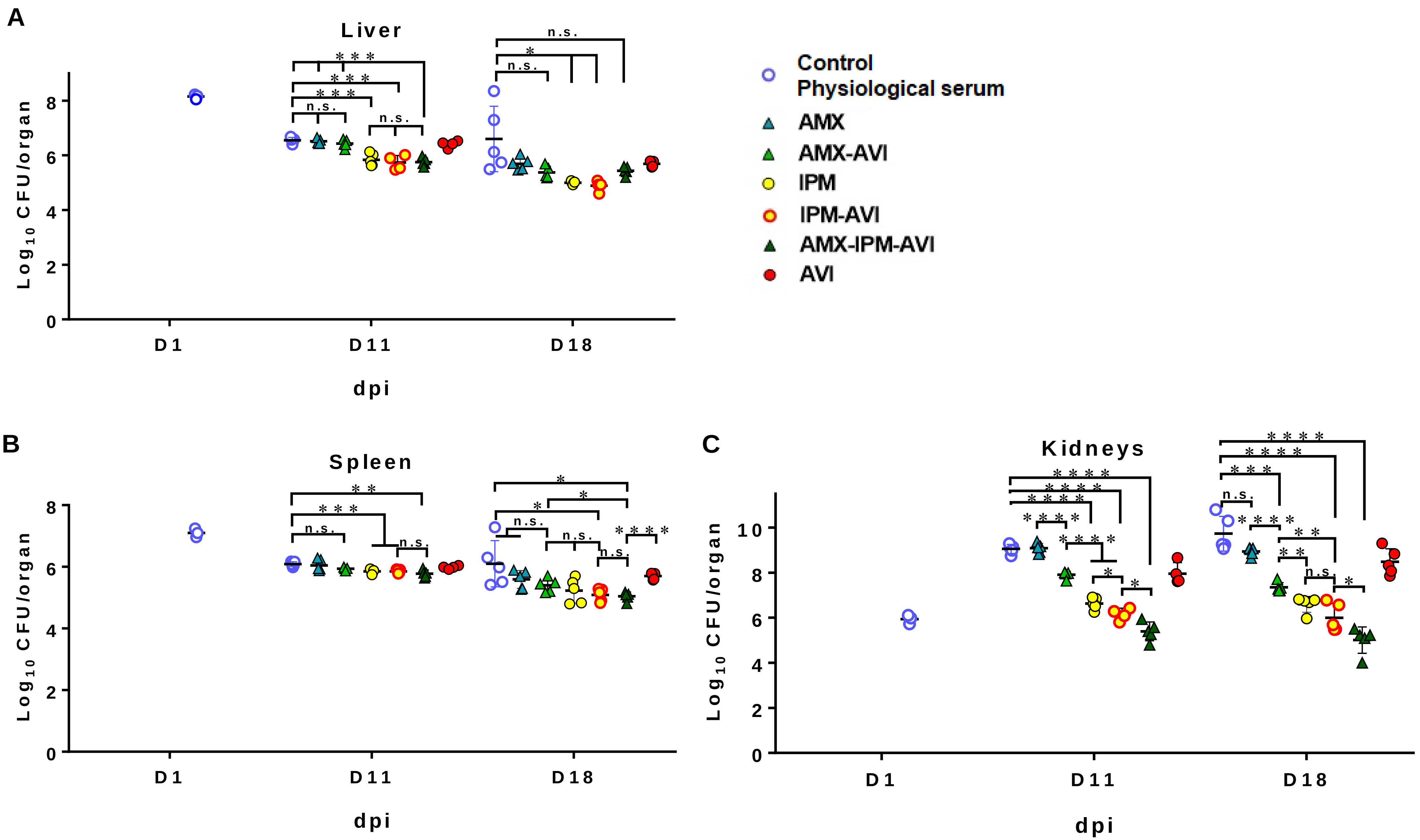

Supplementary Figure 3 : Bacterial persistence in organs of *M. abscessus* smooth variant in C3HeB/FeJ infected mice with treatments as in Fig. 3. *M. abscessus* S-infected C3HeB/FeJ mice untreated (control PBS) or treated with amoxicillin (AMX), the amoxicillin-avibactam combination (AMX-AVI), imipenem (IPM), avibactam (AVI), the combination imipenem-avibactam (IPM-AVI) or the triple amoxicillin-imipenem-avibactam combination (AMX-IPM-AVI). Bacterial persistence of *M. abscessus* CIP 104536^{T} (smooth variant) were measured in the liver (**A**), spleen (**B**) and kidneys (**C**) of C3HeB/FeJ mice after infection and treatment of mice from **Fig. 3**. Differences between means were analyzed by two-way analysis of variance (ANOVA) and the Tukey's post-test, allowing for multiple comparisons. n.s., non-significant; * $P<0.05$, ** $P<0.01$, *** $P<0.001$, **** $P<0.0001$. Experiment was realized once.

Supplementary Table 1
Summary tables of statistical data presented **in Fig. 1 and Fig. S1.**

Supplementary Table 2
Summary tables of statistical data presented in **Fig. 2 and Fig.S2.**

Supplementary Table 3
Summary tables of statistical data presented in **Fig. 3 and Fig. S3.**

Supplementary Table 1

Summary table of statistics from Fig.1 at D10 post-infection

Treatment A \ Treatment B	Control PBS	IPM (100 mg/kg)	IPM-AVI (100 mg/kg)	AVI (100 mg/kg)
Control PBS				
IPM (100 mg/kg)	Lungs: n.s.<br>Liver: *<br>Spleen: n.s.<br>Kidneys: ***			
IPM-AVI (100 mg/kg)	Lungs: n.s.<br>Liver: n.s.<br>Spleen: *<br>Kidneys: **	Lungs: n.s.<br>Liver: **<br>Spleen: n.s.<br>Kidneys: n.s.		
AVI (100 mg/kg)	Lungs: *<br>Liver: n.s.<br>Spleen: *<br>Kidneys: n.s.	Lungs: **<br>Liver: ***<br>Spleen: *<br>Kidneys: ***	Lungs: *<br>Liver: n.s.<br>Spleen: ***<br>Kidneys: **	

n.s. = non-significant,
* *P<0.05*,
** *P<0.01*,
*** *P<0.001*,
**** *P<0.0001*

Summary table of statistics from Fig.1 at D18 post-infection

Treatment A \ Treatment B	Control PBS	IPM (100 mg/kg)	IPM-AVI (100 mg/kg)	AVI (100 mg/kg)
Control PBS				
IPM (100 mg/kg)	Lungs: n.s.<br>Liver: n.s.<br>Spleen: *<br>Kidneys: ***			
IPM-AVI (100 mg/kg)	Lungs: *<br>Liver: **<br>Spleen: **<br>Kidneys: ***	Lungs: n.s.<br>Liver: n.s.<br>Spleen: n.s.<br>Kidneys: n.s.		
AVI (100 mg/kg)	Lungs: n.s.<br>Liver: n.s.<br>Spleen: n.s.<br>Kidneys: *	Lungs: n.s.<br>Liver: n.s.<br>Spleen: n.s.<br>Kidneys: ***	Lungs: n.s.<br>Liver: *<br>Spleen: *<br>Kidneys: **	

n.s. = non-significant,
* *P<0.05*,
** *P<0.01*,
*** *P<0.001*,
**** *P<0.0001*.

Supplementary Table 2

Summary table of statistics from Fig.2 at D11 post-infection

Treatment A \ Treatment B	Control PBS	IPM (100 mg/kg)	IPM-AVI (100 mg/kg)	AVI (100 mg/kg)
**Control PBS**				
**IPM** (100 mg/kg)	Lungs: *<br>Liver: ****<br>Spleen: n.s.<br>Kidneys: ****			
**IPM-AVI** (100 mg/kg)	Lungs: *<br>Liver: ***<br>Spleen: n.s.<br>Kidneys: ****	Lungs: n.s.<br>Liver: n.s.<br>Spleen: n.s.<br>Kidneys: *		
**AVI** (100 mg/kg)	Lungs: n.s.<br>Liver: n.s.<br>Spleen: n.s.<br>Kidneys: n.s.	Lungs: *<br>Liver: ****<br>Spleen: *<br>Kidneys: ****	Lungs: *<br>Liver: ***<br>Spleen: *<br>Kidneys: ****	

n.s. = non-significant,
* *P*<0.05,
** *P*<0.01,
*** *P*<0.001,
**** *P*<0.0001

Summary table of statistics from Fig.2 at D18 post-infection

Treatment A \ Treatment B	Control PBS	IPM (100 mg/kg)	IPM-AVI (100 mg/kg)	AVI (100 mg/kg)
**Control PBS**				
**IPM** (100 mg/kg)	Lungs: **<br>Liver: **<br>Spleen: **<br>Kidneys: ***			
**IPM-AVI** (100 mg/kg)	Lungs: **<br>Liver: **<br>Spleen: **<br>Kidneys: ****	Lungs: n.s.<br>Liver: n.s.<br>Spleen: n.s.<br>Kidneys: *		
**AVI** (100 mg/kg)	Lungs: n.s.<br>Liver: n.s.<br>Spleen: n.s.<br>Kidneys: n.s.	Lungs: ***<br>Liver: ****<br>Spleen: ***<br>Kidneys: ***	Lungs: ***<br>Liver: ****<br>Spleen: **<br>Kidneys: ***	

n.s. = non-significant,
* *P*<0.05,
** *P*<0.01,
*** *P*<0.001,
**** *P*<0.0001.

Supplementary Table 3

Summary table of statistics from Fig.3 at D11 post-infection

Treatment A \ Treatment B	Control PBS	AMX (100 mg/kg)	AMX-AVI (100 mg/kg)	IPM (100 mg/kg)	IPM-AVI (100 mg/kg)	AMX-IPM-AVI (100 mg/kg)	AVI (100 mg/kg)
Control PBS							
AMX (100 mg/kg)	Lungs: n.s.<br>Liver: n.s.<br>Spleen: n.s.<br>Kidneys: n.s.						
AMX-AVI (100 mg/kg)	Lungs: ***<br>Liver: n.s.<br>Spleen: n.s.<br>Kidneys: ****	Lungs: n.s.<br>Liver: n.s.<br>Spleen: n.s.<br>Kidneys: ****					
IPM (100 mg/kg)	Lungs: n.s.<br>Liver: ***<br>Spleen: ***<br>Kidneys: ****	Lungs: n.s.<br>Liver: ***<br>Spleen: n.s.<br>Kidneys: ****	Lungs: n.s.<br>Liver: n.s.<br>Spleen: n.s.<br>Kidneys: ****				
IPM-AVI (100 mg/kg)	Lungs: n.s.<br>Liver: ***<br>Spleen: ***<br>Kidneys: ****	Lungs: n.s.<br>Liver: ***<br>Spleen: n.s.<br>Kidneys: ****	Lungs: n.s.<br>Liver: **<br>Spleen: *<br>Kidneys: ****	Lungs: n.s.<br>Liver: n.s.<br>Spleen: n.s.<br>Kidneys: *			
AMX-IPM-AVI (100 mg/kg)	Lungs: **<br>Liver: ****<br>Spleen: **<br>Kidneys: ****	Lungs: n.s.<br>Liver: ****<br>Spleen: *<br>Kidneys: ****	Lungs: *<br>Liver: ***<br>Spleen: *<br>Kidneys: ****	Lungs: *<br>Liver: n.s.<br>Spleen: n.s.<br>Kidneys: ***	Lungs: *<br>Liver: n.s.<br>Spleen: n.s.<br>Kidneys: *		
AVI (100 mg/kg)	Lungs: n.s.<br>Liver: n.s.<br>Spleen: n.s.<br>Kidneys: n.s.	Lungs: n.s.<br>Liver: n.s.<br>Spleen: n.s.<br>Kidneys: **	Lungs: n.s.<br>Liver: n.s.<br>Spleen: n.s.<br>Kidneys: n.s.	Lungs: n.s.<br>Liver: **<br>Spleen: *<br>Kidneys: **	Lungs: n.s.<br>Liver: **<br>Spleen: *<br>Kidneys: ***	Lungs: ****<br>Liver: ***<br>Spleen: *<br>Kidneys: ****	

n.s. = non-significant, * *P<0.05*, ** *P<0.01*, *** *P<0.001*, **** *P<0.0001*

Summary table of statistics from Fig.3 at D18 post-infection

Treatment A \ Treatment B	Control PBS	AMX (100 mg/kg)	AMX-AVI (100 mg/kg)	IPM (100 mg/kg)	IPM-AVI (100 mg/kg)	AMX-IPM-AVI (100 mg/kg)	AVI (100 mg/kg)
Control PBS							
AMX (100 mg/kg)	Lungs: n.s.<br>Liver: n.s.<br>Spleen: n.s.<br>Kidneys: n.s.						
AMX-AVI (100 mg/kg)	Lungs: n.s.<br>Liver: n.s.<br>Spleen: n.s.<br>Kidneys: ***	Lungs: **<br>Liver: n.s.<br>Spleen: n.s.<br>Kidneys: ****					
IPM (100 mg/kg)	Lungs: **<br>Liver: *<br>Spleen: n.s.<br>Kidneys: ****	Lungs: **<br>Liver: ***<br>Spleen: n.s.<br>Kidneys: ****	Lungs: *<br>Liver: n.s.<br>Spleen: n.s.<br>Kidneys: **				
IPM-AVI (100 mg/kg)	Lungs: **<br>Liver: *<br>Spleen: *<br>Kidneys: ****	Lungs: ***<br>Liver: ***<br>Spleen: *<br>Kidneys: ****	Lungs: **<br>Liver: **<br>Spleen: n.s.<br>Kidneys: **	Lungs: n.s.<br>Liver: n.s.<br>Spleen: n.s.<br>Kidneys: n.s.			
AMX-IPM-AVI (100 mg/kg)	Lungs: **<br>Liver: n.s.<br>Spleen: *<br>Kidneys: ****	Lungs: ****<br>Liver: n.s.<br>Spleen: **<br>Kidneys: ****	Lungs: ***<br>Liver: n.s.<br>Spleen: *<br>Kidneys: ****	Lungs: *<br>Liver: ***<br>Spleen: n.s.<br>Kidneys: ***	Lungs: *<br>Liver: n.s.<br>Spleen: n.s.<br>Kidneys: *		
AVI (100 mg/kg)	Lungs: n.s.<br>Liver: n.s.<br>Spleen: n.s.<br>Kidneys: n.s.	Lungs: n.s.<br>Liver: n.s.<br>Spleen: n.s.<br>Kidneys: n.s.	Lungs: *<br>Liver: n.s.<br>Spleen: *<br>Kidneys: **	Lungs: **<br>Liver: ****<br>Spleen: *<br>Kidneys: ***	Lungs: **<br>Liver: ****<br>Spleen: ***<br>Kidneys: ***	Lungs: **<br>Liver: *<br>Spleen: ****<br>Kidneys: ****	

n.s. = non-significant, * *P<0.05*, ** *P<0.01*, *** *P<0.001*, **** *P<0.0001*.
